# High-Throughput Assay for the Identification of Compounds Regulating Osteogenic Differentiation of Human Mesenchymal Stromal Cells

**DOI:** 10.1371/journal.pone.0026678

**Published:** 2011-10-26

**Authors:** Hugo Alves, Koen Dechering, Clemens Van Blitterswijk, Jan De Boer

**Affiliations:** 1 Department of Tissue Regeneration, MIRA Institute for Biomedical Technology and Technical Medicine, University of Twente, Enschede, The Netherlands; 2 Department of Molecular Pharmacology, Merck Research Laboratories, Oss, The Netherlands; Massachusetts Institute of Technology, United States of America

## Abstract

Human mesenchymal stromal cells are regarded as the golden standard for cell-based therapies. They present multilineage differentiation potential and trophic and immunosuppressive abilities, making them the best candidate for clinical applications. Several molecules have been described to increase bone formation and were mainly discovered by candidate approaches towards known signaling pathways controlling osteogenesis. However, their bone forming potential is still limited, making the search for novel molecules a necessity. High-throughput screening (HTS) not only allows the screening of a large number of diverse chemical compounds, but also allows the discovery of unexpected signaling pathways and molecular mechanisms for a certain application, even without the prior knowledge of the full molecular pathway. Typically HTS is performed in cell lines, however, in this manuscript we have performed a phenotypical screen on more clinically relevant human mesenchymal stromal cells, as a proof of principle that HTS can be performed in those cells and can be used to find small molecules that impact stem cell fate. From a library of pharmacologically active small molecules, we were able to identify novel compounds with increased osteogenic activity. These compounds allowed achieving levels of bone-specific alkaline phosphatase higher than any other combination previously known. By combining biochemical techniques, we were able to demonstrate that a medium to high-throughput phenotypic assay can be performed in academic research laboratories allowing the discovery of novel molecules able to enhance stem cell differentiation.

## Introduction

Osteoporosis and other bone-related disorders represent a major public health threat. One out of every two women and one in four men, aged 50 or older, is expected to develop an osteoporosis-related fracture in their lifetime [1], [2], [3]. There is a huge demand for products enhancing bone regeneration. Therefore, the last decade was denominated the bone and joint decade by the World Health Authority. Over the last years, hMSCs have become the golden standard for cell therapy applications, mainly due to their multilineage potential, but also due to their secretion of trophic and immunomodulatory factors [4], [5].

However, bone tissue engineering using hMSCs is being hindered by the lack of markers predicative of bone formation and due to the relatively poor performance of hMSCs, and there is therefore an urgent need for new molecules able to induce their differentiation potential with higher efficiency. In the past we and others have tried different candidate approaches in order to enhance the performance of hMSCs for bone tissue engineering applications, including the effect of Wnt signaling on the proliferation and differentiation of hMSCs [6], the effect of the inhibition of histone deacetylases on mineralization [7], and the activation of the cAMP/PKA pathway [8]. New technology is currently available in research laboratories, allowing the discovery of unexpected signaling pathways and molecular mechanisms for a certain application by screening libraries of reagents such as small molecules, siRNA or peptides.

High-throughput screening (HTS) is a process that allows the screen of thousands of chemicals in order to identify potential interesting compounds for a specific application. HTS techniques have been used for some years, but the technical settings and especially the costs involved in its implementation have restricted their application to large pharmaceutical companies. More recently, this method has become more accessible and, therefore, it is currently a technique that can be performed in an academic setting. In pharmacology, HTS assays are usually developed based on a certain target molecule in a cell line which is easy to culture. However, for bone tissue engineering, the usage of this technology for phenotypical screens in clinically relevant cells would be more appropriate since it would allow the discovery of new compounds and possible new target molecules for cell-based bone tissue engineering. Using phenotypical assays, entire pathways of interest can be discovered, providing the opportunity for multiple intervention points, as opposed to a single direct molecular target commonly used in biochemical approaches. Cell-based assays can therefore be used to identify modulators of differentiation pathways (for example osteogenesis) in the physiological environment of the cell with all the intact regulatory networks and feedback control mechanisms present. The possibility to combine compounds is almost unlimited and several libraries should be explored. For bone tissue engineering, several approaches can be undertaken including the screening of libraries of small compounds (as described in this manuscript), the possibility to screen libraries of biomaterials generated by combinatorial chemistry [9] and libraries of surface topographies [10].

Although the design of the screens can vary, compound screens are usually performed at single dosage and a single measurement for each compound in the initial screen. This is frequently the only option available to economically screen a large library of compounds. Compounds identified are then re-tested and then further evaluated at different dosages. After a compound is validated by several techniques, it is usually considered as a lead and then can be further tested as a potential drug candidate for future clinical trials. Several aspects have to be taken into consideration while designing a screen, and both false negatives and false positives should be minimized by avoiding or correcting systematic errors [11], [12], [13]. Frequently a cutoff is established based on statistics to affirm positive compounds.

Alkaline phosphatase (ALP) is currently the most frequently used marker for osteogenic differentiation and it has been previously used as a readout in the search for novel osteogenic suppressors in hMSCs [14], novel promoters and inhibitors of osteogenic differentiation [15], and in the assessment of the osteogenic capacity of several compounds [8], [16], [17].

Based on our experience in bone tissue engineering, we sought to find novel osteogenic molecules by performing a phenotypical screening of a library of pharmaceutically active compounds and, therefore, we describe a simple, yet effective way of screening for compounds in a common research laboratory without the need of expensive robotic techniques.

## Materials and Methods

### Isolation, culture and characterization of hMSCs

Bone marrow aspirates were obtained from donors with written informed consent and were approved by the medical ethical committee of the University medical Centre Utrecht. Briefly, aspirates (Table S1) were resuspended using 20 G needles, plated at a density of 5×10^5^ cells/cm^2^ and cultured in hMSC proliferation medium (PM) containing α-minimal essential medium (α-MEM, Life Technologies), 10% fetal bovine serum (FBS, Cambrex), 0.2 mM ascorbic acid (Asap, Life Technologies), 2 mM L-glutamine (Life Technologies), 100 U/ml penicillin (Life Technologies), 10 µg/ml streptomycin (Life Technologies) and 1 ng/ml basic fibroblast growth factor (bFGF, Instruchemie). Cells were grown at 37°C in a humid atmosphere with 5% CO_2_. Medium was refreshed twice a week and cells were used for further subculturing or cryopreservation when reaching 80–90% confluence. After expansion, cells were characterized for surface marker expression (CD73, CD90, CD 105, CD11b, CD19, CD34, CD45 and HLA-DR) as previously described [18]. Furthermore, their multilineage potential was confirmed by performing several differentiation experiments [18]. hMSC basic medium/control medium (BM) was composed of hMSC PM without bFGF. hMSC osteogenic medium (OM) was composed of hMSC basic medium supplemented with 10^−8^ M dexamethasone (dex, Sigma).

### LOPAC library for the phenotypical screen

**Figure 1:**
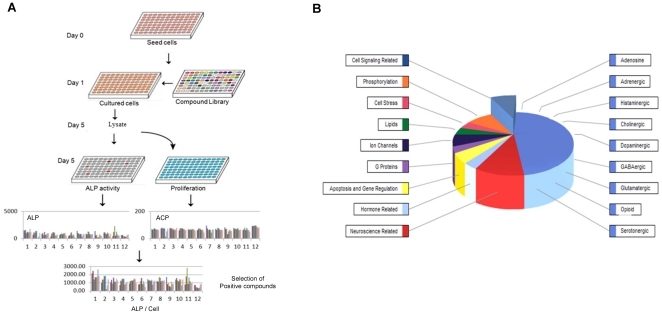
High-throughput Assay. **A**) Schematic representation of HTA: hMSCs were grown on proliferation medium, dissociated using trypsin and plated into 96 well plates at a density of 2000 cells per well in osteogenic medium. Cells were allowed to attach for 24 h, after which medium was refreshed and compounds and controls were added to each plate. After 4 days, both ALP activity and proliferation was measured in a fluorescence plate reader. The compounds activity was evaluated in relation to the controls used. Positive compounds were then further confirmed by FACS and taken for further analysis. **B**) Representative diagram of the classes of action of the compounds used for the screen (Lopac library, Sigma-Aldrich).

The Lopac library (Sigma-Aldrich) was purchased to screen for osteogenic compounds. It is composed of 1280 pharmacologically active compounds, with all multiple targets represented including GPCRs, kinases and ion channels (see Figure 1B for a representative diagram of the classes of action of the compounds used on the screen). Many molecules are marketed drugs and have pharmaceutically relevant structures, with predictable activity and directed against a wide range of drug targets. The compounds are highly purified, and the stock is pre-solubilized in DMSO. The final compound concentration used in the screen was 4,5 µM in a volume of 200 µL per well of osteogenic medium (OM), containing 0.25% DMSO (v/v). In each test plate columns were reserved for positive (OM+0.25% DMSO) and negative (BM+0.25% DMSO) controls.

### High-throughput assay (HTA)

To assess the osteogenic potential of the compounds present in the HTA library, hMSCs were seeded at 2000 cells/cm^2^ in BM and allowed to attach overnight. The next day, medium was changed to OM and test compounds and controls were added to the plates. This initial screen was performed in hMSCs from two different donors (D36 and D41) to cover possible donor variation in the osteogenic response. After 4 days of incubation in OM, relative cell number was determined by measuring acidic phosphatase (ACP) activity [19]. First, cells were washed twice with PBS (Life technologies) and lysed using 30 µL of 0.2% Triton X-100 solution in 100 mM PBS pH 7.8, supplemented with a protease inhibitor cocktail (Roche Applied Science). For the measurement of cell numbers, 5 µL of the lysate was incubated with 100 µL of 5 mM 4-nitrophenyl phosphate disodium salt, dissolved in 0.1 M sodium acetate, 0.1% Triton-X, pH 5.5 for 1.5 hours at 37°C. After the incubation period, the reaction was stopped by adding 10 µL of 0.5 N sodium hydroxide and allowed to equilibrate for 10 min. The absorbance was then measured at 405 nm.

Alkaline phosphatase activity was measured using a biochemical ALP assay. For this assay, part of the previously described lysate was incubated in the dark at 25°C with CDP-Star substrate (Roche) and allowed to react for 30 min. Luminescence was then measured using a VICTOR^3^ luminometer (Perkin Elmer) at 25°C. The total ALP luminescence was normalized for cell number using the ACP activity as readout.

### Primary Screen validation and Statistic analysis

In order to measure the assay quality we used the most widely accepted method, the Z′ factor [20]. This metric quantifies the separation of a positive activity (positive control - OM) from the background (negative control – BM) in the absence of test compounds. To determine this factor the following formula was used:
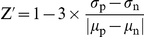
where σ_p_ and σ_n_ are the standard deviations of the positive and negative control, respectively, and μ_p_ and μ_n_ are their means. A score of Z′≥0.5 indicates and excellent assay, which is able to discriminate positive hits from background noise.

The next step involved the selection of a meaningful cutoff on the calculated statistics to declare positive compounds. Since it was only possible to test 80 compounds per plate and since positive and negative hits could easily influence the plate sample mean and standard deviation, to establish the cutoff we calculated the standard deviations from the trimmed mean of the tested compounds. The top and bottom 10% of the tested compounds were not taken into account for the calculation of the mean and standard deviation and hits were selected if they were higher than 3 SD-away from the trimmed mean and were present in at least 2 independent screens.

### Hit validation

The hit compounds obtained from the primary screen were then re-evaluated at 10 different concentrations in order to obtain the optimal concentration that induced the highest osteogenic activity. All the compounds were serially diluted in half-logarithmic steps, ranging from 3.16e^−9^ M to 1e^−4^ M. hMSCs were seeded at 2000 cells/ cm^2^ in triplicate in both basic and osteogenic medium for 4 days. After this period, cells were lysed and both cell number and ALP activity were calculated as previously described. The optimal concentration was then selected for further studies.

Next, we confirmed enhanced ALP expression as a secondary screen by flow cytometry. Cells were seeded at 5000 cells/cm^2^ in basic medium (BM), allowed to attach for 10–15 h and then cultured for 4 days in both BM and OM. Each experiment consisted of a negative control (cells grown in BM), a positive control (cells grown in OM), and one or more experimental conditions. Experiments were performed in triplicate and at least 10000 cells were measured. After 4 days of treatment, cells were trypsinized and incubated for 30 min. in phosphate-buffered saline solution (PBS) containing 5% bovine serum albumin (BSA), after which cells were further incubated in PBS-1% BSA containing primary antibody (anti-ALP B4-78; Developmental Studies Hybridoma Bank, University of Iowa, Iowa City, IA) for 1 hr. Cells were then washed and incubated with secondary antibody (goat anti-mouse IgG conjugated with phycoerythrin [PE]) for 30 min. After incubation, cells were washed three times and resuspended in PBS-1% BSA. Viaprobe (BD Biosciences) was added for live/dead staining and allowed to incubate for 10 min. Cells were then analyzed using a BD FACScan (BD Biosciences) and ALP levels were determined on live cells only.

## Results

### Assay Development

When developing an hMSC-based assay there are several critical steps, being reproducibility and assay validation the most important ones. A high-throughput assay was established as shown in Figure 1. On day 0, hMSCs were dissociated using trypsin and a single cell suspension was obtained. hMSCs were then seeded in 96-well plates at 1000 cells/well in basic medium (BM) for one day, which allowed them to attach and recover better from the trypsinisation process. Medium was then changed to osteogenic medium (OM) and a library of 1280 active compounds were tested for 4 days. This period of time was considered optimal since it did not require re-addition of compounds and change of medium. For the selection of positive compounds, we used a luminescence-based ALP activity assay, and ACP activity to correct for cell number. This combination of assays works well and was compared with others such as CellQuant and para-nitrophenyl phosphate (pNPP) based-ALP measurement, yielding similar results, but reducing significantly the timeframe and workload (data not shown). In order to validate the assay, the Z score was calculated for each plate individually and for a control plate. The values ranged from 0.6 to 0.7, meaning that the HTA is able to discriminate possible hits from statistical noise on a given plate. With this methodology we achieved a reliable and relatively fast method to screen libraries of compounds with osteogenic potential.

### Screening of the LOPAC library

In order to investigate new molecules with osteogenic potential, high-throughput assays of a library of 1280 pharmacologically active compounds were performed in hMSCs from 2 donors (D36 and D41). The compounds were screened at a single dosage of 4.5 µM in a volume of 200 µL per well of osteogenic medium (OM), containing 0.25% DMSO (v/v). ALP levels were normalized for cell number, and profound cytotoxicity was taken as an exclusion criterion, since for bone tissue engineering, proliferation is crucial in order to achieve proper differentiation. Only the compounds that were able to induce ALP above 3 SD-away from the trimmed mean of its plate and did not present a significant reduction of proliferation were taken into account. These hits were then compiled and only the ones appearing in more than one screen (on both donors tested) were taken for further studies. In the initial screens, 29 and 32 hits were obtained, from which 14 were present in both screens.

With ACP as readout, we have assessed whether any compounds exerted a mitogenic effect, and although some compounds did enhance significantly the proliferation of certain donor cells, there were no compounds that were able to significantly enhance proliferation in both donors tested, possible due to the fact that the mitogenic effect of the compounds was donor-specific.

### Selection of the optimal concentration

**Figure 2:**
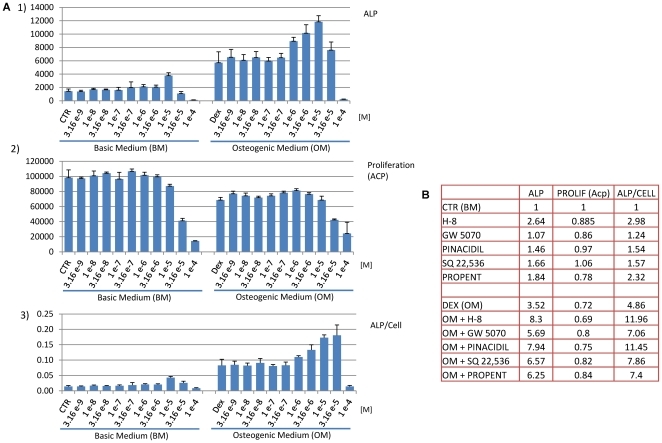
Hit validation. After being selected from the primary screen, the identified compounds (hits) were subjected to half-logarithmic dilutions in order to assess the optimal concentration of each compound that would induce the highest alkaline phosphatase (ALP) activity. The graphs represent: **A1**) ALP activity; **A2**) ACP activity **A3**) ALP activity/cell, in both basic and osteogenic medium when supplemented with different concentrations of the compound H-8 (as example, since the same was performed for all the hits obtained); **B**) The table represents the ALP-induction ratio when compared to the control situation (basic medium).

After the compounds were selected from the primary screen, a subsequent dose-response curve was established in both basic and osteogenic medium. From the 14 compounds obtained in the initial screen, 2 were not available for retesting. An example of this assay for one compound (H-8) can be seen in Figure 2A. When H-8 is added to basic medium, at a concentration of 1 µM, an induction of ALP activity(≈2×) can be seen (Figure 2A1), lower concentrations do not produce any effect and higher ones present a decrease in total ALP activity. This decrease in ALP activity can be explained by a profound decrease in cell proliferation at concentrations higher than 3.16 µM (Figure 2A2). The same phenomenon occurs in osteogenic medium where dexamethasone is present, however a synergistic effect can already be seen for concentrations as low as 1 µM. Although adding 3.16 µM of H-8 to the medium induces a higher ALP activity per cell than 1 µM (Figure 2A3), the latter was chosen for further studies, since the former presents significant cytotoxicity (Figure 2A2). The same concentration range was used for all the different compounds and the optimal concentration obtained for each compound was then used for further studies. From the 12 compounds tested, we confirmed 5 (Figure S1). The induction ratios (ALP activity, proliferation and ALP per cell) of the compounds validated at the optimal concentration were compared to the control situation (basic medium) and are represented in Figure 2B. The compounds that induced higher ALP activity were H8 and Pinacidil when used in combination with dexamethasone.

### Hits Validation by flow cytometry

**Figure 3:**
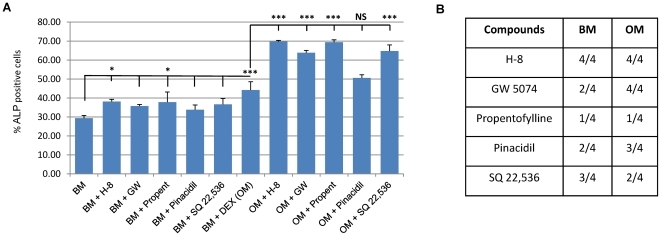
Confirmation of the Hits by flow cytometry on ALP. **A**) Evaluation of the osteogenic potential of several treatment conditions by flow cytometry on hMSCS after 4 days treatment. Data is expressed as percentage of ALP positive cells. At least 10,000 cells were measured for each condition and experiments were performed in triplicate. Error bars represent standard deviation. Statistical analysis was performed using (one-way ANOVA and Tukey post-test with a significance level of 0.05. Asterisks represent * P<0.05, ** P<0.01 and *** P<0.001. BM (basic medium); OM (Osteogenic Medium); DEX (Dexamethasone). **B**) Number of donors in which the respective compound had a positive effect on ALP induction both in basic or osteogenic medium.

**Figure 4:**
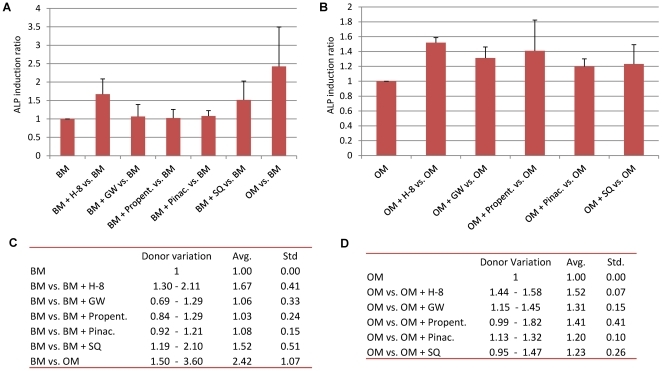
Confirmation of the hits by flow cytometry in 3 extra donors. **A,B**) Evaluation of the osteogenic potential of several treatment conditions on hMSCS after 4 days. Data is expressed as ALP induction ratio. Data reflects the average of induction of the percentage of ALP-positive cells of each condition divided by the average of induction of the percentage of ALP-positive cells of the respective control (A – basic medium and B – osteogenic medium). Each experimental condition was performed in triplicate and data reflects the results of 3 independent donors). **C,D**) Table depicting the donor variation on the induction of the percentage of ALP positive cells after each experimental condition when compared to the respective control.

After the compounds were assessed for the optimal dosage to induce the highest ALP activity (marker for bone formation) without interfering significantly with proliferation, they were further validated in hMSCs derived from 4 donors (D26, D38, D41 and D42) by flow cytometry, using an antibody against bone/liver/kidney-specific ALP. The percentage of cells expressing the ALP marker was evaluated and all the conditions were compared to either basic or osteogenic medium. Figure 3 represents the data from D41 (one of the donors used in the initial screen (Figure 3A), and since some donor variation was present, we also include a table that summarizes the number of donors where each compound was effective (Figure 3B). In the cells from D41 (A), we can observe that in control medium, the maximum effect of a compound in basic medium (BM) was an increase from 29.37%±1.44 of ALP positive cells (BM) to 38.12%±1.20 (when H-8 was added to BM). However, it is clear that the best effect obtained by any compound is achieved when osteogenic medium (OM) is used. While the compound with less effect in this donor (Pinacidil) is able to synergistically increase the % ALP positive cells from 44.19%±4.44 (OM) to 50.62%±1.63, others (H-8 and Propentofylline) can significantly increase it up to 69.76%±0.68 and 69.46%±1.30, respectively, representing a 57% increase over dexamethasone (the reference osteogenic molecule). However, not all the compounds exhibited a consistent effect on the four donors tested (Figure 3B). Only one compound (H-8) was able to induce ALP in both BM and OM in the four donors tested, while 2 compounds (H-8 and GW 5074) were able to induce it in OM medium. The compound with the mildest effect was Propentofylline, since it only increased ALP in one donor (D41), although in that particular donor, was able to induce it significantly (from 44.19±4.44% of positive cells to 69.46%±1.30, representing a≈57% increase over dex. alone) (Figure 4A). In this donor, except for Pinacidil, all the other compounds that were used in OM, were able to significantly enhance ALP levels when compared to Dexamethasone alone (OM), showing that they were able to synergistically enhance ALP expression. When used alone (BM) only H-8 and Propentofiline were able to significantly induce ALP (Figure 3A).

An overview of the average induction level (for 3 extra donors D26, D38 and D42) of each compound tested compared to the control can be seen in Figure 4. For each condition, data represents the average of the percentage of ALP positive cells for the three donors tested and was divided by the average of the controls to achieve the induction ratio. As can be seen from Figure 4A, the compound that induces most ALP, in basic medium, is dexamethasone (positive control) with an average of 2.42 times more ALP-positive cells than the control. However the induction ratio varied largely between donors (1.50 to 3.69) as seen by the standard deviation (Figure 4A) and the data present in Figure 4C. From the compounds obtained in the screen, only H-8 and SQ 22,536 induced significantly more ALP positive cells than the control. However, when the compounds were added to OM (Figure 4B), all compounds induce an average ALP expression higher than the control (OM), showing that they synergistically act with dexamethasone to enhance ALP. The donor variation observed is depicted in Figure 4C (BM) and 4D (OM).

### Comparison of the validated hits with the reference osteogenic molecules

**Figure 5:**
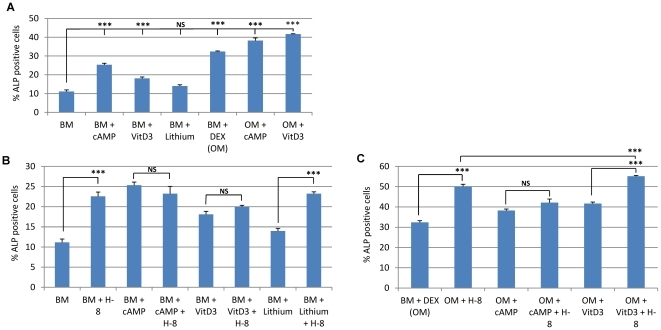
Hit compound activity in the context of reference osteogenic compounds. The current graphs represents only one of the compounds validated (H-8) and all the possible combinations with the reference osteogenic molecules. Graphs represent the % ALP-positive cells of: the controls (**A**), the different combinations possible in BM (**B**) and the different combinations in OM (**C**). The osteogenic potential of each treatment was assessed by FACS after 4 days, in at least 10000 cells, in triplicate. Error bars represent standard deviation. Statistical analysis was performed using one-way ANOVA and Tukey post-test with a significance level of 0.05. Asterisks represent * P<0.05, ** P<0.01 and *** P<0.001. OM – basic medium supplemented with dexamethasone; cAMP – di-buteryl cyclic AMP, DEX – dexamethasone, NS – non significant.

After performing the validation of the hits, we tested their activity in the context of other known compounds with known osteogenic activity (Figure 5). *N* ^6^, 2′-*O*-dibutyryladenosine 3′,5′-cyclic monophosphate sodium salt (cAMP) was used as a reference osteogenic molecule since we have previously shown that PKA pathway activation (by cAMP) in hMSCs *in vitro* results in robust bone formation *in vivo* [*8*]. The highest percentage of ALP positive cells was obtained when reference molecules vitamin D3 and dexamethasone (41.67%±0.22) were combined (OM+VitD3) (Figure 5A). This combination yielded a significantly higher percentage of ALP-positive cells than the combination of cAMP and dexamethasone (p<0.01), or dexamethasone alone (p<0.001). Supplementing this combination (OM+VitD3) with H-8 increased the percentage of ALP positive cells even further to 55.16%±0.77, representing an approx. 13% increase (Figure 5C). However, the addition of H-8 alone to OM already induced 50.13%±0.46 of cell to express ALP (Figure 5C). Even in the basic medium, the addition of H-8 was able to increase the percentage of ALP-positive cells by 50%, from 11.15%±0.85 (BM) to 22.56%±1.06 (BM+H-8) (Figure 5B). The addition of H-8 to known osteogenic compounds (Dexamethasone, VitD3) increased ALP levels in a synergistic way (Figure 5C), except with cAMP where the changes induced by the addition of H-8 were not significant. Since lithium is known to inhibit the osteogenesis via Wnt signaling in hMSCs, we also tested the effect of lithium to H-8-induced ALP expression. The addition of lithium did not have any effect on H-8-induced ALP (Figure 5B), suggesting that the ALP induction by H-8 was not inhibited by the Wnt signaling pathway.

### General Overview

**Figure 6:**
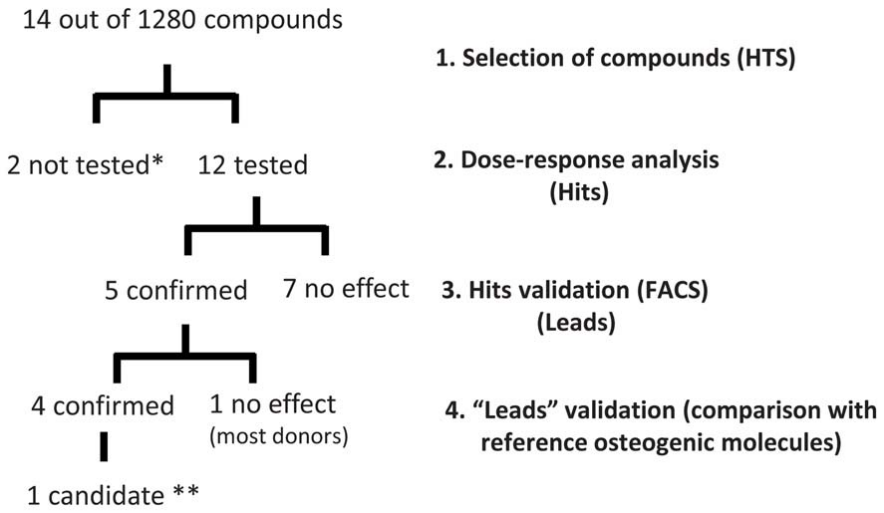
Results from the Lopac library screen. Diagram showing the results obtained from the 1280 compounds library screen. The evaluation process was composed of 4 main steps. **1.** Selection of the compounds from the primary screen compared to the controls. **2.** Dose-curve evaluation of the optimal concentration to be used (fluorescence-based read out). **3.** Confirmation by FACS of the hits in cells from 3 other donors. **4.** Confirmation of the osteogenic potential and comparison to the reference osteogenic compounds known so far. Out of 1280 compounds, 14 were identified in the primary screen, which were then subjected to dose-response analysis resulting in 5 hits. From these 5 hits, 4 were confirmed by FACS to have enhanced osteogenic potential in most donors. These 5 compounds (leads) were then further evaluated in 3 other donors and compared to reference osteogenic compounds which have resulted in novel osteogenic combinations with increased osteogenic potential that were never tested before. * Compounds not tested were discontinued from the manufacturer or had problems with solubility and therefore were not in conformation with the manufacturers specifications. ** Candidate molecule for *in vivo* studies, proven to induce osteogenesis in all donors tested and had a synergistic effect with reference osteogenic molecules.

A general overview of the results is described in Figure 6. From the total 1280 compounds, 14 were selected for subsequent dose-response assays, in order to find the optimal dose to induce ALP. From the 14 selected compounds, 2 were not possible to be tested, and from the 12 that were assayed, 5 exhibited dose-depend effects on ALP induction. The list of the 5 compounds can be found in Figure S1. The hits were then tested further by flow cytometry on three other donors, which resulted in 4 compounds with consistent ALP induction above the controls (Dexamethasone) and one (Propentofylline) that presented a non-significant increase in ALP expression in most donors. Based on this, H-8 is the most promising compound for future *in vivo* studies.

## Discussion

### Design of the High-throughput assay

When designing an HTA/ HTS the first consideration should be the goal of the screen and then the type of assays that would allow achieving that goal with the highest efficiency. Since our primary goal was to search for novel molecules with high osteogenic activity, the type of cells and readout marker to be used was our first consideration. Concerning cells, there were two immediate possible choices available, each one with their advantages and drawbacks. We had to choose whether we would use a highly homogeneous and abundant cell line with osteogenic potential (such as C2C12 or MC3T3) or use human primary cells derived from bone marrow biopsies. While the first ones provide a more homogeneous cell source which will possibly result in an increased reproducibility between assays, they are isolated from mouse and are immortalized cell lines. Therefore, the results obtained could not directly been extrapolated to the human situation, without previously take in consideration, the species difference and even the differentiation state of the cells. An example is the usage of cAMP as an osteogenic inducer, where we have shown that the same compound results in an opposite effect when used in human cells as opposed to rodent models [21]. This study stresses the need that research experiments should be performed in more clinically relevant models.

Human mesenchymal stromal cells do not present the problem of species variation, however they are more heterogeneous, less abundant and present donor variation [22]. Nevertheless, when care is taken in the isolation of the cells and in the design of the experiment, most of these factors can be overcome. We therefore fell that the use of human primary cells is more clinically relevant and although more cumbersome since cells from different donors have to be used, it also contributes for the strengthening and validation of the results for possible future clinical applications. Furthermore, since HMSCs are multipotent, they could be a useful tool to unveil compounds that influence cell fate decisions. In this manuscript, we show that some compounds that induce osteogenesis are donor-specific, while others are able to robustly induce in all donors tested, making them better suited for *in vivo* studies.

For a fast and efficient high-throughput screening several factors are needed, one of them being the choice of the assays used as readout. They should be fast, economic, efficient, reproducible and, in the case of HTS, they should be simple enough to allow automation. The first step was the choice of assay to be used to determine cell numbers. Several methods exist, either by the direct or indirect determination of cell number, including cell counting using a microscope or a particle counter [23], determination of cellular protein or DNA [24], [25], [26], measurement of 3H-thymidine incorporation [25], measurement of the uptake and conversion of tetrazolium dyes such XTT, MTS, and MTT [27], [28], [29], [30], or by measuring cellular enzymes such as lactic dehydrogenase [31]. However, most of them fail to obey to the desired characteristics mentioned above especially because most of them are time-consuming, therefore offering limited use in large-scale screening experiments. Another major limitation is that most of these methods are not sensitive enough to determine very low numbers of cells (less than 1000) and simple enough to be automated. When analyzing the effect of drugs on cell proliferation, one has to realize that these compounds can often be cytotoxic and therefore, it may be that in some wells the amount of cells is limited, therefore, high-sensitivity is a prerequisite. Although, the assays based on cellular uptake and reduction of tetrazolium dyes are considered by some authors as the quickest, simplest, and most sensitive of the methods mentioned above, they offer several limitations [27]. These limitations include the fact that dye-based assays frequently suffer from high background signals, lack of reproducibility and limited linear response, in addition to the fact that some human cells metabolize tetrazolium dyes very inefficiently, not to mention that in some cases such dyes are even toxic to cells. In our screen we choose to use a well-established method to detect the activity of acidic phosphatase, due to the several advantages that this method offers on comparison to others. It is a simple method, it allows automation, can be performed in frozen lysates, it is inexpensive, reproducible and sensitive enough to detect low cell numbers. Acidic phosphatase activity assays can be performed since the endogenous levels of this enzyme is relatively constant in many cell types under different culture conditions and these methods have been proven to be the most sensitive and reproducible for counting different cell sources [19].

For the ALP activity quantification there was the possibility of quantifying it by using pNPP as substrate (colorimetric) or a luminescence method based on the conversion of the substrate CDP-Star (Roche). CDP-Star is a chemiluminescent substrate for alkaline phosphatase that enables an extremely sensitive and fast detection of biomolecules. It generates a luminescence signal which is approximately 10 times stronger than comparable chemiluminescent substrates and the signal is stable for long time. This method is fast, sensitive, presents very high signal to noise ratio and it is easily automated. After careful optimization and validation we decided to use this method for the detection of ALP activity.

Since the compounds were dissolved in DMSO the initial concern was to assess the potential toxicity of the dosage to be used. By reducing the amount of DMSO used in relation to the total volume of medium we achieved a very low final DMSO concentration (0.25%) reducing significantly DMSO cytotoxicity (data not shown). Controls were used in each plate to assess for DMSO effects and also to provide a reference for the compounds being tested. We also opted to change medium in the plates only before the addition of the compounds present in the library. Thus, we used the compounds of the library only once, reducing the costs in both compounds and materials, in addition to the labour needed to replace the old medium and add new compounds. It also decreased the potential effect of adding DMSO for a second time, and since we previously knew that ALP was expressed at higher levels already after 4 days, we opted for the convenience of the screen, to use this time frame for the assay.

### Hit Rate on HTS screens and advantages of phenotypical screens

One of the biggest problems in high-throughput screening experiments is the amount of false positive hits (inactive compounds misidentified as active by the primary screen). The concept of hit-rate designates the ratio of the number of actives to the number of compounds screened. Hit rates vary largely amongst screens and depend on several factors for instance whether the screen uses unknown synthetic compounds or “known bioactives”. Despite known bioactive libraries are typically much smaller than commercial libraries of unknown synthetic compounds, they typically have a significantly higher hit rates than the libraries of synthetic compounds [32]. In our screen of bioactive compounds, 14 hits from a total of 1280 compounds tested were obtained, which corresponded to 1,09% of all compounds tested. Then on the counter screen (the dose-curve experiment) only 5 of the initial hits were confirmed and therefore, effectively, only 0,39% of the total screened compounds went for further validation. These hit rates are in line with other high-throughput screens (HTS), and as an example, in the siRNA library screen for osteogenic suppressors the hit rate was around 1,06% [14], while in another HTS based on ALP as readout the hit rate for promoters of osteogenesis was around 3,46% [15]. The percentage varies largely among bioactive screens, and in some cases the hit rates can be as high as 53,8%, as was the case of a screen for activators of deacetylase activity of purified Sirt1, where 1,151 initial hits were obtained out of 2,139 molecules screened (ChemBank). Without prior consideration of the true number of active compounds present in a library for the application in study, one cannot foresee the number of hits expected to be obtained in the first screen.

### Validated hits and their mechanism of action

The compounds selected as hits do not induce osteogenesis via the same signaling pathways, however, most of them are somehow involved in either the Raf-MEK-ERK or the cAMP signaling pathway. This discovery is in line with our previous findings that cAMP can mediate osteogenesis [8].

The most promising compound from the screen that was proven to enhance ALP formation in all the donors tested is H-8, which has been described as a potent, cell-permeable, reversible and ATP-competitive inhibitor of cyclic-nucleotide protein kinases. It is known to inhibit protein kinase A(PKA), myosin light chain kinase (MLCK), protein kinase C (PKC) and protein kinase G (PKG) [33]. We previously reported, and show in Figure 5, that cAMP induces ALP expression, which is mediated via the activation of protein Kinase A (PKA). Yet, H-8 is still able to enhance significantly the ALP levels, apparently independent of PKA activity. The mechanism by which this occurs is still unknown. As mentioned, the mechanism of action of H-8 is broader than PKA alone. Similarly, a number of studies have identified that the action of 2 other PKA inhibitors (H89 and KT 5720) are independent of their effects on PKA. Low specificity of small molecules is a widespread phenomenon and include actions on other protein kinases and signaling molecules and also on basic cellular functions, such as transcription [34].

Interestingly, the other compound that was also able to increase the dex-induced ALP in all the tested donors was GW 5074, which is a Raf 1 kinase inhibitor, immediately downstream of Ras in the MAPK signaling pathway [35]. Amongst other effects, it stimulates Raf-MEK-ERK pathway and enhances the effects of most HDAC inhibitors [36].

The third compound more effective in OM was Pinacidil, which is a K_ATP_ channel opener, known to hyperpolarize various cell types. Both Pinacidil and other K_ATP_ channel openers like Diazoxide, are known to elevate bone marker expression, namely BSP and ALP, and it was shown that endogenous hyperpolarization is a functional determinant of hMSC differentiation and a possible control point for modulating stem cell function [37], [38].

The other compounds selected as hits are both involved in the cAMP signaling however their effect seems to be somehow antagonist, which might lead to the speculation that their effects could be broader than the cAMP pathway itself. One of these compounds is SQ 22,536. It is a cell-permeable adenylate cyclase (AC) inhibitor [39] and since AC is the main responsible for the transformation of ATP to cyclic AMP (cAMP), the addition of SQ 22,536 should result in a decrease in cAMP levels, which is in contradiction with our previously published data [8] but in line with the results obtain with the compound H-8 and with the report on the siRNA library screen for osteogenic suppressors [14]. However as a note, this compound was only effective in half of the donors tested and when all the donors are taken into account the addition of SQ 22,536 presented an average higher ALP than OM, but this increase was not significantly different. It could be that the increase on ALP observed could be via other pathway than the cAMP one, since it was recently proven that many compounds involved in cAMP pathway have a broader action than what was previously known.

The last compound is Propentofylline. It is a cAMP phosphodiesterase inhibitor, an adenosine transport inhibitor and a non-selective adenosine receptor antagonist. It has been proven to decrease free radical formation and lipid peroxidation [40], to reinforce the cAMP signaling and enhance the cAMP levels in plasma of mice [41]. Its predominant mechanism of action appears to be causally related to an increase of tissue cAMP/cGMP mainly by a) an indirect reinforcement of A2 receptor mediated cAMP formation (via adenosine transport blockade) and b) hindrance of intracellular cAMP breakdown (via inhibition of cAMP- and cGMP-PDEs). As a note, this compound was only active in one of the donors used to validate the screen results, but in this particular donor, the increase in ALP expression was approx. 57% more than dexamethasone alone, which was highly significant. However this effect appeared to be donor specific.

Despite some of these compounds interact in pathways that are known to control osteogenesis, the full mechanism of action of some is still unknown. In phenotypical screens, however, the effect of a compound on the phenotype of choice, in this case osteogenic differentiation, is the relevant read-out. Even without detailed molecular knowledge on the mechanism of action of compounds, the positive effect of them can be exploited. Nevertheless, further work needs to be performed to deepen the knowledge of the signaling pathways investigated and should be regarded as an extra tool to reveal potential drug targets for future manipulation.

### Conclusions

In this manuscript we show that by performing an HTA we were able to obtain compounds that are able to enhance ALP expression (the best known indicator of osteogenic differentiation) even when compared to the reference osteogenic molecules (Dexamethasone, Vitamin D3, cAMP). The results obtained by this HTA show that many more compounds have the potential to induce ALP, and prove that this methodology can be very effective in discovering novel compounds with osteogenic potential or novel functions to currently known compounds. Furthermore, this type of HTA can be easily extrapolated to other differentiation experiments, once the methodology is changed.

In this screen only a relatively small library of compounds was tested (1280 compounds) and therefore many other libraries can reveal compounds that are still not known to induce osteogenesis. What would be equally interesting and highly valuable for the research in bone formation, would be the discovery of bone formation markers that are able to predict bone formation more efficiently, making the HTS with those markers even a more valuable tool towards improved bone formation and hopefully making the transition from bench to bedside quicker.

## Supporting Information

Figure S1 **Selected hits from the initial screen.** Compilation of the compounds selected and their respective molecular formula and known function.
(TIF)

Table S1 **Donor List.** Compilation of the different donors whose hMSCs were used on the various experiments of this manuscript.
(TIFF)
