# Supplementary figures and images for: High-Throughput Assay for the Identification of Compounds Regulating Osteogenic Differentiation of Human Mesenchymal Stromal Cells
Source: PLoS One. 2011 Oct 26;6(10):e26678. doi: 10.1371/journal.pone.0026678 (PMC3202560; doi:10.1371/journal.pone.0026678)

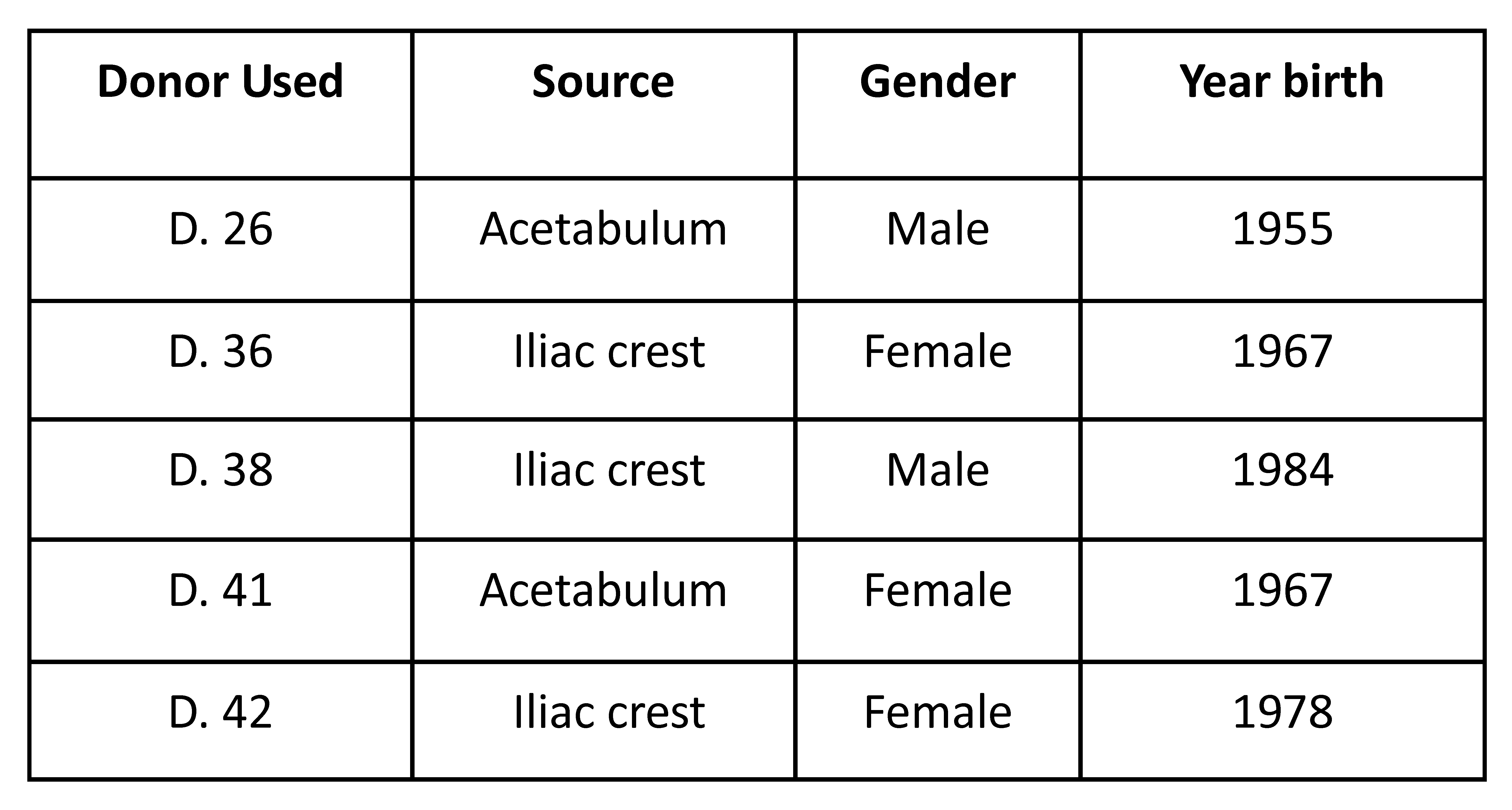

Supplement: Table S1 — Donor List. Compilation of the different donors whose hMSCs were used on the various experiments of this manuscript. (TIFF) [file pone.0026678.s002.tiff]
